# The gut microbiota and metabolome are associated with diminished COVID-19 vaccine-induced antibody responses in immunosuppressed inflammatory bowel disease patients

**DOI:** 10.1016/j.ebiom.2022.104430

**Published:** 2023-01-10

**Authors:** James L. Alexander, Benjamin H. Mullish, Nathan P. Danckert, Zhigang Liu, Marton L. Olbei, Aamir Saifuddin, Melissa Torkizadeh, Hajir Ibraheim, Jesús Miguéns Blanco, Lauren A. Roberts, Claire M. Bewshea, Rachel Nice, Simeng Lin, Hemanth Prabhudev, Caroline Sands, Verena Horneffer-van der Sluis, Matthew Lewis, Shaji Sebastian, Charlie W. Lees, Julian P. Teare, Ailsa Hart, James R. Goodhand, Nicholas A. Kennedy, Tamas Korcsmaros, Julian R. Marchesi, Tariq Ahmad, Nick Powell

**Affiliations:** a Division of Digestive Diseases, Department of Metabolism, Digestion and Reproduction, Faculty of Medicine, Imperial College London, London, United Kingdom; b Department of Gastroenterology and Hepatology, Imperial College Healthcare NHS Trust, London, United Kingdom; c St Mark's Hospital and Academic Institute, Harrow, London, United Kingdom; d King's College London, London, United Kingdom; e University of Exeter, Exeter, Devon, United Kingdom; f Department of Gastroenterology, Royal Devon and Exeter NHS Foundation Trust, Exeter, Devon, United Kingdom; g National Phenome Centre, Department of Metabolism, Digestion and Reproduction, Faculty of Medicine, Imperial College London, London, United Kingdom; h Hull University Teaching Hospitals NHS Trust, Gastroenterology, Hull, United Kingdom; i University of Hull, Hull York Medical School, Hull, United Kingdom; j Western General Hospital, Edinburgh, United Kingdom; k The University of Edinburgh Centre for Genomic and Experimental Medicine, Edinburgh, United Kingdom; l Earlham Institute, Norwich, United Kingdom; m Quadram Institute Bioscience, Norwich, United Kingdom

**Keywords:** Gut microbiota, Metabolome, SARS-CoV-2, Inflammatory bowel disease, Anti-TNF therapy, Infliximab, Vaccine, ChAdOx1 nCoV-19, BNT162b2, COVID-19

## Abstract

**Background:**

Patients with inflammatory bowel disease (IBD) treated with anti-TNF therapy exhibit attenuated humoral immune responses to vaccination against SARS-CoV-2. The gut microbiota and its functional metabolic output, which are perturbed in IBD, play an important role in shaping host immune responses. We explored whether the gut microbiota and metabolome could explain variation in anti-SARS-CoV-2 vaccination responses in immunosuppressed IBD patients.

**Methods:**

Faecal and serum samples were prospectively collected from infliximab-treated patients with IBD in the CLARITY-IBD study undergoing vaccination against SARS-CoV-2. Antibody responses were measured following two doses of either ChAdOx1 nCoV-19 or BNT162b2 vaccine. Patients were classified as having responses above or below the geometric mean of the wider CLARITY-IBD cohort. 16S rRNA gene amplicon sequencing, nuclear magnetic resonance (NMR) spectroscopy and bile acid profiling with ultra-high-performance liquid chromatography mass spectrometry (UHPLC-MS) were performed on faecal samples. Univariate, multivariable and correlation analyses were performed to determine gut microbial and metabolomic predictors of response to vaccination.

**Findings:**

Forty-three infliximab-treated patients with IBD were recruited (30 Crohn's disease, 12 ulcerative colitis, 1 IBD-unclassified; 26 with concomitant thiopurine therapy). Eight patients had evidence of prior SARS-CoV-2 infection. Seventeen patients (39.5%) had a serological response below the geometric mean. Gut microbiota diversity was lower in below average responders (p = 0.037). *Bilophila* abundance was associated with better serological response, while *Streptococcus* was associated with poorer response. The faecal metabolome was distinct between above and below average responders (OPLS-DA R^2^X 0.25, R^2^Y 0.26, Q^2^ 0.15; CV-ANOVA p = 0.038). Trimethylamine, isobutyrate and omega-muricholic acid were associated with better response, while succinate, phenylalanine, taurolithocholate and taurodeoxycholate were associated with poorer response.

**Interpretation:**

Our data suggest that there is an association between the gut microbiota and variable serological response to vaccination against SARS-CoV-2 in immunocompromised patients. Microbial metabolites including trimethylamine may be important in mitigating anti-TNF-induced attenuation of the immune response.

**Funding:**

JLA is the recipient of an NIHR Academic Clinical Lectureship (CL-2019-21-502), funded by 10.13039/501100000761Imperial College London and The Joyce and Norman Freed Charitable Trust. BHM is the recipient of an NIHR Academic Clinical Lectureship (CL-2019-21-002). The Division of Digestive Diseases at Imperial College London receives financial and infrastructure support from the 10.13039/100014461NIHR Imperial Biomedical Research Centre (BRC) based at 10.13039/501100000762Imperial College Healthcare NHS Trust and 10.13039/501100000761Imperial College London. Metabolomics studies were performed at the MRC-NIHR National Phenome Centre at Imperial College London; this work was supported by the 10.13039/501100000265Medical Research Council (MRC), the 10.13039/100014461National Institute of Health Research (NIHR) (grant number MC_PC_12025) and infrastructure support was provided by the NIHR Imperial Biomedical Research Centre (BRC). The NIHR Exeter Clinical Research Facility is a partnership between the University of Exeter Medical School College of Medicine and Health, and Royal Devon and Exeter NHS Foundation Trust. This project is supported by the National Institute for Health Research (NIHR) Exeter Clinical Research Facility. The views expressed are those of the authors and not necessarily those of the NIHR or the UK Department of Health and Social Care.

**Research in context**

**Evidence before this study**

Patients with inflammatory bowel disease (IBD) treated with anti-TNF therapy exhibit attenuated humoral immune responses to vaccination against SARS-CoV-2. However, even in anti-TNF recipients, vaccine-induced antibody responses are highly heterogeneous. Such variability is not explained by known modifiers of vaccine response including age, vaccine type or concomitant immunomodulator use, indicating that other, undefined factors influence vaccine immunogenicity. The gut microbiota and its functional metabolic output, which are perturbed in IBD, play an important role in shaping host immune responses. There have been very few studies exploring associations between the gut microbiota and immune response to vaccination, although a causal link has been drawn between depletion of the microbiota, reduction in secondary bile acids and attenuated response to seasonal influenza vaccination.

**Added value of this study**

In the current study, we explore whether the gut microbiota and metabolome can explain variation in anti-SARS-CoV-2 vaccination responses in immunosuppressed IBD patients treated with anti-TNF. The key findings are that gut microbiota diversity is lower in below average responders to vaccination. *Bilophila* abundance is associated with better serological response, while *Streptococcus* is associated with poorer response. The faecal metabolome is distinct between above and below average responders. Trimethylamine (TMA) and isobutyrate are associated with better response, while succinate, phenylalanine and the bile acids taurolithocholate and taurodeoxycholate are associated with poorer response.

**Implications of all the available evidence**

To our knowledge, this study is the first to demonstrate a link between the metabolic function of the gut microbiota and immune responses to SARS-CoV-2 vaccination in a vulnerable clinical group. Our data indicate that therapeutics targeted at modulating the microbiota, or even supplementation of beneficial metabolites such as TMA, might be effective strategies to ameliorate diminished vaccine-induced immunogenicity in vulnerable groups.

## Introduction

Vaccination against SARS-CoV-2 is an effective strategy to limit infections, hospitalisations and deaths caused by COVID-19.^1^^,^^2^ However, SARS-CoV-2 vaccine immunogenicity is diminished in some immunosuppressed groups. Patients with inflammatory bowel disease (IBD) treated with anti-TNF therapy have attenuated serological responses to vaccination against SARS-CoV-2, which is associated with increased risk of breakthrough infection.^3^, ^4^, ^5^, ^6^ However, even in anti-TNF recipients, vaccine-induced antibody responses are highly heterogeneous. Such variability is not explained by known modifiers of vaccine response including age, vaccine type or concomitant immunomodulator use,^3^ indicating that other, undefined factors influence vaccine immunogenicity.

The gut microbiota is critical in shaping host immune responses, and microbiota function has been proposed as a potential modulator of response to vaccination. In infants, faecal microbiota composition is associated with responses to oral rotavirus vaccines.^7^^,^^8^ In adults given a broad-spectrum antibiotic intervention prior to vaccination against influenza,^9^ reduced H1N1-specific neutralization and IgG1 and IgA binding antibody titres were observed. Lower titres were correlated with both bacterial and metabolomic phenotypes and, in particular, a 1000-fold reduction in serum secondary bile acids. Impaired antibody responses to influenza vaccine have been shown in antibiotic-treated, germ-free and *Tlr5*^*-/-*^ mice compared to littermate controls.^10^ Such an effect was also seen with inactivated poliovirus, but not with other vaccines against Hepatitis B and yellow fever.^10^ The ability of faecal microbiota transplant to rescue impaired immune responses to several vaccines has also been demonstrated in preclinical models.^11^ Recent studies of recipients of SARS-CoV-2 vaccination have shown associations between gut microbiota composition and short chain fatty acid (SCFA) levels with neutralising antibody responses in non-immunosuppressed healthy volunteers.^12^^,^^13^

Inflammatory bowel disease is associated with perturbation of gut microbiota composition and function.^14^^,^^15^ Microbiota composition has also been linked to therapeutic responses to immunosuppressive therapies in IBD,^16^ and gut microbial metabolites are predictive of response to anti-TNF therapy.^17^^,^^18^ Given the three-way interaction between IBD, the gut microbiota and immunosuppressive therapy, we conducted a prospective observational study to explore the hypothesis that gut microbiota composition and function influence immune response to SARS-CoV-2 vaccination in immunosuppressed patients with IBD.

## Methods

### Recruitment and sampling

Patients with IBD established on infliximab treatment for greater than 12 weeks were recruited at two centres (Imperial College Healthcare NHS Trust and London North West University Healthcare NHS Trust). All participants were in the CLARITY-IBD study (https://www.clarityibd.org/), ≥18 years old and undergoing vaccination against SARS-CoV-2. Participants were excluded at screening if they had received antibiotics within 6 weeks of recruitment. All recruited subjects provided written informed consent.

Faecal and blood samples were collected from patients either when they attended hospital infusion units to receive infliximab, or on the day they attended the hospital vaccine centre to receive their first dose of anti-SARS-CoV-2 vaccine. The majority of faecal samples were collected shortly before the first dose of vaccine was given and 81% of faecal samples were collected within 4 weeks of the first dose. Blood samples were collected at 8-week intervals according to the CLARITY-IBD study protocol (available at https://www.clarityibd.org).

**Fig. 1:**
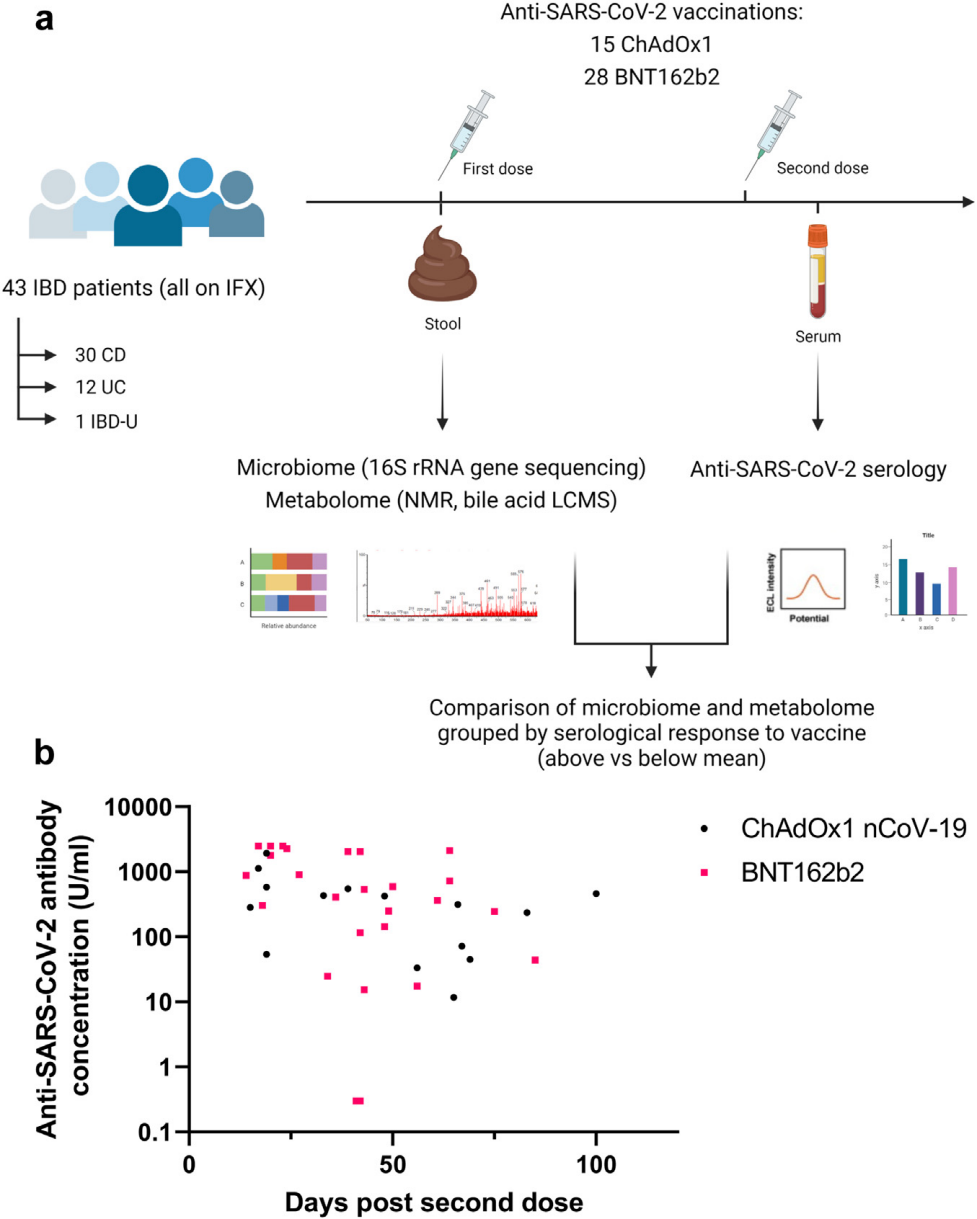
(a) Experimental schema for the study; (b) Anti-SARS-CoV-2 spike RBD antibody concentrations in patients receiving ChAdOx1 nCoV-19 (black dots) or BNT162b2 (pink squares). Each point represents a single patient plotted the number of days after second dose of vaccine when their antibody level was measured.

Whole faeces were collected in a faeces collector (FECOTAINER®, AT Medical BV, The Netherlands). Fresh faecal samples were transferred to the laboratory within 6 hours of collection, homogenized and aliquoted for storage at −80 °C pending analysis. An experimental schema is shown in Fig. 1a.

### Anti-SARS-CoV2 serology

Anti-SARS-CoV-2 serology laboratory analyses were performed at the Academic Department of Blood Sciences at the Royal Devon and Exeter NHS Foundation Trust. To determine antibody responses specific to vaccination we used the Roche Elecsys Anti-SARS-CoV-2 spike (S) electrochemiluminescence immunoassay.^19^ This double sandwich electrochemiluminescence immunoassay uses a recombinant protein of the receptor binding domain (RBD) on the spike protein as an antigen for the determination of antibodies against SARS-CoV-2. Sample electrochemiluminescence signals are compared with an internal calibration curve and quantitative values are reported as units (U)/mL. In-house validation experiments have been described previously.^3^

All participants were tested for previous SARS-CoV-2 infection using the Roche Elecsys anti-SARS-CoV-2 (N) immunoassay. A concentration of greater than or equal to 0.12 U/ml was defined as a threshold below which participants were deemed to have no evidence of prior infection. Participants who reported a previous PCR test confirming SARS-CoV-2 infection at any time prior to vaccination were deemed to have evidence of past infection irrespective of any antibody test result.

All participants in the study had serological analyses at 8-week intervals, irrespective of when their vaccine doses were given. Thus, serology results were available for participants at varying time points. The CLARITY-IBD study generated rolling geometric mean data for infliximab-treated patients with and without history of prior SARS-CoV-2 infection. These data demonstrate that the geometric mean antibody concentration was higher in patients with prior infection, and antibody concentrations waned over time. To standardise our analysis and account for the effects of prior infection and varying time between second vaccine dose and serological analysis, we took the anti-S RBD antibody concentration at the first time-point measured post-second vaccine dose (but no earlier than 14 days after the second dose). This concentration was compared to the geometric mean at the relevant time point as measured in the infliximab-treated CLARITY-IBD cohort (specific to vaccine-type and presence/absence of prior natural infection). Participants were classified as having serological responses above or below their vaccine-type, infection-specific geometric mean.

### 16S rRNA gene sequencing

DNA was extracted from crude faecal samples using the DNeasy PowerLyzer PowerSoil Kit (Qiagen, Hilden, Germany) following manufacturer's instruction with the modification that samples were homogenized in a Bullet Blender Storm bead beater (Chembio, St Albans, UK). DNA was quantified using a Qubit Fluorometer (ThermoFischer, UK), and was aliquoted and stored at −80 °C until ready for downstream use. Sample libraries were prepared following Illumina's 16S Metagenomic Sequencing Library Preparation Protocol^20^ using specifically designed V1/V2 hypervariable region primers.^21^ Pooled sample library sequencing was performed using the Illumina MiSeq platform (Illumina Inc, Saffron Walden, UK) and the MiSeq Reagent Kit v3 (Illumina) using paired-end 300-bp chemistry. Processing of sequencing data was performed via the DADA2 pipeline (v1.18) as previously described,^22^ using the SILVA bacterial database Version 138.1 (https://www.arb-silva.de/ (accessed on 08/10/2021)). Pre-processed assigned sequence variants (ASVs) data were further cleaned and filtered using the decontam pipeline removing contaminating DNA features.^23^ The ASV dataset was trimmed with a 10% threshold (i.e. ASVs present in fewer than 10% of samples were not included).

### Metabolomic profiling using ^1^H NMR

Faecal metabolite extraction was performed as previously published with modification.^24^ 600 mg of each faecal sample was mixed with 1200 μl water, vortexed for 5 min and spun at 20,000 g for 10 min at 4 °C. 540 μl supernatants were collected and mixed with 60 μl phosphate buffer containing 1.5 M KH_2_PO_4_, 1 mM NaN_3_ and 1‰ TSP. 570 μl of the mixtures were transferred into a 5 mm NMR tube for analysis. One-dimensional proton NMR spectra was acquired using a Bruker 600 MHz spectrometer (Bruker, Rheinstetten, Germany) at 300 K employing a standard NOESY pulse sequence (relaxation delay - 90° - t1 - 90° - tm - 90° - acquisition). Relaxation delay was set to 4 s, while t1 and tm were set to 4 μs and 100 ms, respectively. 32 scans were performed into 64 K data points with a spectral width of 20 ppm. Fourier transformation, phase and baseline correction and calibration of TSP at δ 0.0 of NMR spectra were carried out in Topspin V3.0 software (Bruker Biospin). Data were imported into MATLAB R2014a (MathWorks Inc., Natick, USA) with a resolution of 0.0005 ppm for further processing. After the removal of the water peak (δ 4.7–4.9) and TSP peak (δ -1 – 0.6), the spectra were automatically aligned using the recursive segment-wise peak alignment (RSPA) algorithm to reduce peak shifting effects,^25^ and also normalized by the probabilistic quotient method to diminish inter-sample concentration variability.^26^ Metabolites were assigned using Chenomx NMR Suite 8.31 (Chenomx Inc., Edmonton, Canada) and published literature.^24^ Statistical Total Correlation Spectroscopy (STOCSY) analysis was employed to assist the metabolite identification.^27^

### Bile acid profiling using UHPLC-MS

Faecal samples were analysed using ultra high-performance liquid chromatography-mass spectrometry (UHPLC-MS) for the profiling of bile acids. The protocols used for faecal extract preparation and sample acquisition were as previously described.^21^^,^^28^^,^^29^ In brief, 75 μL aliquots were taken from each sample and diluted 1:3 v/v with water including a mixture of stable isotope-labelled internal standards added in order to monitor data quality during acquisition. To assess retention time drift (and for subsequent bile acid annotation) mixes of bile acid standard references were also acquired as part of the analysis run. For quality control (QC) and pre-processing, a pooled QC sample was prepared by combining equal parts of each study sample and acquired at regular intervals during sample analyses. An additional set of QC sample dilutions was created and analysed at the start and end of sample analyses for assessment of analyte response.^30^

Sample analysis was performed on an ACQUITY UPLC instrument (Waters Corp., Milford, MA, USA) coupled to Xevo G2-S Q-TOF mass spectrometers (Waters Corp., Manchester, UK) via a Z-spray electrospray ionisation (ESI) source operating in negative ion mode. Target bile acids were extracted using peakPantheR, an automated pipeline for the detection, integration and reporting of predefined metabolites from LC-MS data.^31^ The resulting dataset was pre-processed using the nPYc-Toolbox.^32^ Pre-processing included elimination of potential run-order effects and feature filtering to retain only features/metabolites measured with a high analytical quality (relative standard deviation, RSD in pooled QC<30%, pooled QC dilution series Pearson correlation to dilution factor>0.7, RSD in study samples>1.1∗ RSD in pooled QC). Using these techniques, the relative abundances of 50 bile acid species were obtained.

### Statistical analysis

A combination of R packages was used to analyse and visualise faecal microbiota sequencing data, including Phyloseq,^33^ Vegan,^34^ and ggplot 2.^35^ To assess feature changes in alpha-diversity, mixed effects models^36^ compared Shannon's diversity index and Chao 1 richness with the average antibody concentration (i.e. above or below geometric mean, categorical) adjusting for covariates (age, vaccine-type, IBD subtype, hospital centre, immunomodulator use, gender, ethnicity, body mass index (BMI) and comorbidity). Aitchison distance was used for beta-diversity analyses^37^ after centre log-ratio data transformation (CLR)^38^; principal coordinates analyses (PCoA) were generated to visualise the (dis)similarity between groups. Permutational multivariate analysis of variance (PERMANOVA) and Permutation Test for Constrained Correspondence Analysis, Redundancy Analysis and Constrained Analysis of Principal Coordinates were used to statistically compare groupings within the data.^34^ Differential abundance analysis of ASVs was calculated using ANCOM-BC^39^ with Holm-Bonferroni p-value correction and a mixed effects model (adjusting for aforementioned covariates) with false discovery rate (FDR) correction.^36^

^1^H NMR and UHPLC-MS data were analysed using SIMCA (v17) and R (v4.0.3). After Pareto scaling of ^1^H NMR data, unsupervised Principal Components Analysis (PCA) and supervised Orthogonal Projections to Latent Structures Discriminant Analysis (OPLS-DA) were performed with further interrogation of data using S plot and validation of supervised models using CV-ANOVA. Mixed effects models^40^ assessed metabolite differences between groups with differentially abundant metabolites reported with q value < 0.2 after correction for multiple testing (Benjamini-Hochberg false discovery rate). Finally, Spearman correlation of statistically significant ASVs with metabolites was performed using corrplot (q < 0.2 considered significant after false discovery rate p value correction).

### Network analysis

The filtered Spearman correlation matrices were converted into weighted edge lists and used to generate undirected correlation networks with the R language package igraph. The resulting networks were further processed and visualized with Cytoscape (version 3.8.2),^41^ where the degree of nodes was calculated using the analyze network option.

### Ethical considerations

Human samples used in this research project were obtained from the Imperial College Healthcare Tissue Bank (ICHTB). ICHTB is supported by the National Institute for Health Research (NIHR) Biomedical Research Centre based at Imperial College Healthcare NHS Trust and Imperial College London. ICHTB is approved by Wales REC3 to release human material for research (17/WA/0161). All participants were co-recruited from the CLARITY-IBD study (20/HRA/3114).

### Role of funding source

No funding sources had any role in the writing of the manuscript or the decision to submit it for publication. The authors were not precluded from accessing data in the study, and they accept responsibility to submit for publication.

## Results

Forty-three patients with IBD were recruited between 25th January and 15th March 2021. Demographics are shown in Table 1.

**Table 1:** Participant characteristics (total n = 43).

Characteristics	N
Male:Female	27:16
Median age (range)	40 (19–67)
Median BMI (IQR)	24.8 (22.1–27.3)
Ethnicity	
*White*	33 (76.7%)
*Asian*	6 (13.9%)
*Mixed*	2 (4.7%)
*Other*	2 (4.7%)
Vaccine:	
*Oxford/AstraZeneca(ChAdOx1 nCoV-19)*	15
*Pfizer/BioNTech(BNT162b2)*	28
IBD therapy:	
*Infliximab*	43 (100%)
*Thiopurine*	26 (60.5%)
IBD subtype:	
*Crohn's disease*	30
*Ulcerative colitis*	12
*IBD-unclassified*	1
Comorbidity	
*Heart disease*	0 (0%)
*Diabetes mellitus*	0 (0%)
*Lung disease*	5 (11.6%)
*Kidney disease*	0 (0%)
Prior SARS-CoV-2 infection	8 (18.6%)
Median faecal calprotectin (IQR)	196 (56–485)

### Anti-SARS-CoV-2 (S) antibody level following two doses of COVID-19 vaccine

Anti-SARS-CoV-2 spike antibodies were measured between 14 and 100 days after the second dose of vaccination (Fig. 1b). Across the cohort, 25 patients (58.1%) had antibody levels above the geometric mean, specific to their vaccine type and prior infection status; 18 patients (41.9%) had antibody levels below the geometric mean. There were no significant differences in the proportions of patients above and below the geometric mean when stratified by age, IBD subtype, vaccine type, immunomodulator use or patient gender (supplementary Fig. 1a–e).

### Gut microbiota composition is associated with response to anti-SARS-CoV-2 vaccination

**Fig. 2:**
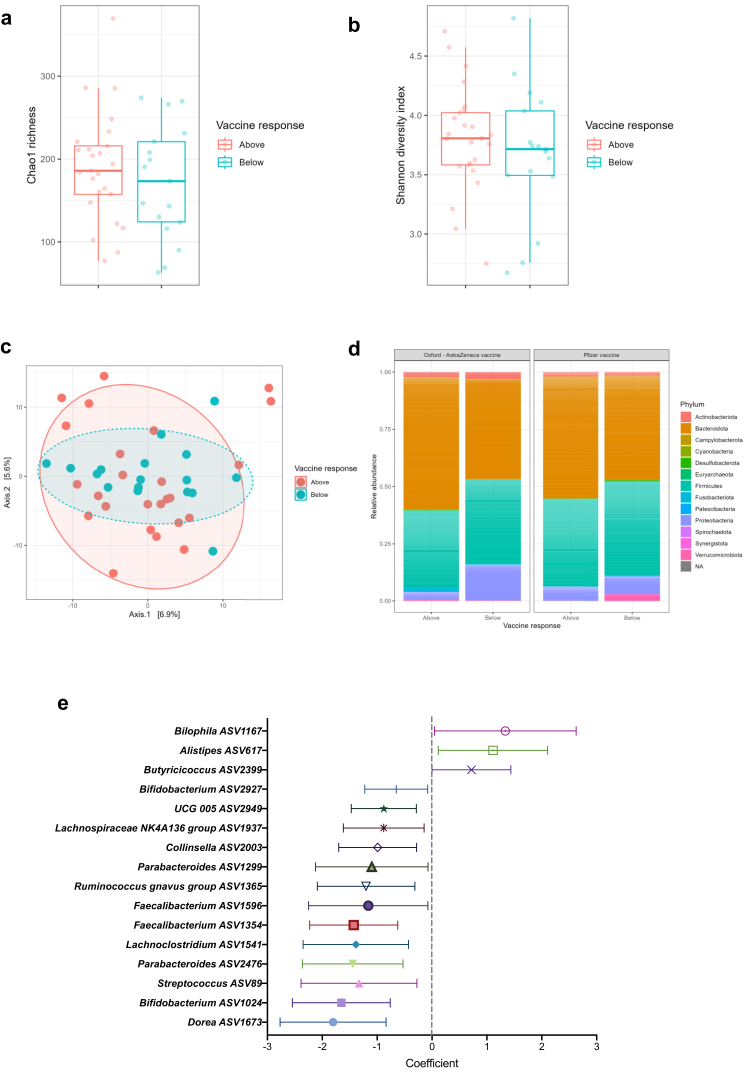
**Gut microbiota composition associated with serological response to two doses of anti-SARS-CoV-2 vaccine.** (a) Chao 1 and (b) Shannon Diversity metrics, showing no difference in alpha diversity between above (red) and below (blue) average vaccine responders (Kruskal–Wallis tests p = ns); (c) Principal coordinates analysis (PCoA) plot of Aitchison's distances in above (red) and below (blue) average responders (numbers on points represent study number; Permutation Test for Constrained Correspondence Analysis, Redundancy Analysis and Constrained Analysis of Principal Coordinates p = 0.037; PERMANOVA test p = ns); (d) Phylum level relative abundance bar plots for above and below average responders to Oxford/Astra-Zeneca (ChadOx-nCoV-19) vaccine (left) and Pfizer/BioNTech (BNT162b2) vaccine (right). (e) Univariate analysis of ASVs identified as differentially abundant between above and below average vaccine responders. Points indicate the coefficients and horizontal error bars indicate the 95% confidence intervals.

Baseline gut microbiota composition was found to differ between vaccine recipients with above average antibody concentrations compared to those with below average concentrations. Although there were no significant differences in Chao 1 or Shannon alpha diversity metrics between the groups (Fig. 2a and b), nor on Permutational multivariate analysis of variance (PERMANOVA), beta diversity analysis demonstrated reduced dispersion of inter-sample Aitchison distances in the below average group compared to the above average group (Fig. 2c; Permutation Test for Constrained Correspondence Analysis, Redundancy Analysis and Constrained Analysis of Principal Coordinates p = 0.037). The dominant phyla in above and below average responders to both the BNT162b2 and the ChAdOx1 nCoV-19 vaccines were *Bacteroidota* and *Firmicutes* (Fig. 2d). The proportion of *Bacteroidota* was lower in below average responders to both vaccines, although this was not statistically significant (Fig. 2d). After accounting for covariates including IBD subtype, vaccine type, immunomodulator use, patient age, gender, BMI, ethnicity, comorbidity and hospital centre, a total of sixteen ASVs were differentially abundant between above and below average responders (Fig. 2e). In particular, *Bilophila* (coefficient 1.34 [95% CI 0.04–2.63])*, Alistipes* (1.11 [0.11–2.11]) and *Butyricicoccus* (0.72 [0.004–1.44]) were associated with above average response. Thirteen ASVs were associated with below average response, including *Streptococcus* (−1.33 [−2.39 to −0.28]) and *Parabacteroides* (−1.45 [−2.36 to −0.53]).

### Gut metabolome is associated with response to anti-SARS-CoV-2 vaccination

**Fig. 3:**
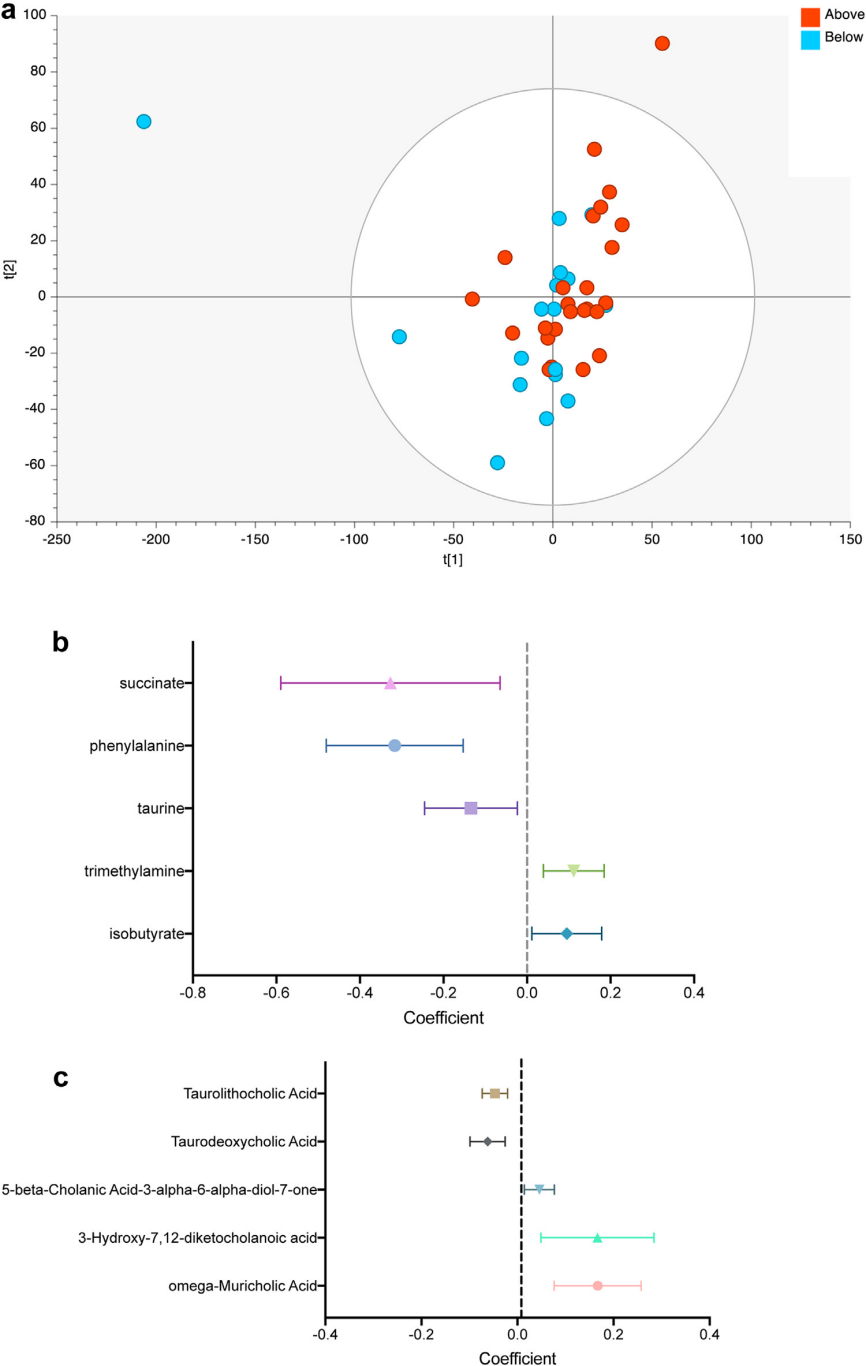
**Faecal metabolomics show associations between gut microbial function and serological response to vaccination**. (a) Principal Components Analysis showing clustering of participants based on ^1^H NMR profile (2 PCs, R^2^ = 0.42, Q^2^ 0.12; OPLS-DA model (not shown) R^2^X 0.25, R^2^Y 0.26, Q^2^ 0.15; CV-ANOVA p = 0.038). (b) Univariate analysis of metabolites identified as differentially abundant between above and below average vaccine responders in ^1^H NMR. (c) Univariate analysis of bile acids identified as differentially abundant between above and below average vaccine responders in UPLC-MS. Points indicate the coefficients and horizontal error bars indicate the 95% confidence intervals.

^1^H NMR profiles were first used to interrogate global metabolite profiles in above and below average responders. In multivariate analysis, the faecal metabolome of above average responders to vaccination was distinct from that of below average responders (Fig. 3a; PCA R^2^X 0.42, Q^2^ 0.12; OPLS-DA model R^2^X 0.25, R^2^Y 0.26, Q^2^ 0.15, CV-ANOVA p = 0.038). A total of 36 metabolite features were identified from ^1^H NMR, including five short chain fatty acids, eleven amino acids and three respiratory compounds. 50 bile acids were assigned with targeted UHPLC-MS. In univariate analysis (Fig. 3b–c), higher levels of trimethylamine (coefficient 0.11 [95% CI 0.04–0.18]), isobutyrate (0.10 [0.01–0.18]) and omega-muricholic acid (0.17 [0.08–0.26]) were found in the faecal metabolomes of patients with above average responses to vaccination. Below average vaccine response was characterised by abundance of succinate (coefficient −0.33 [95% CI −0.59 to −0.06]), phenylalanine (−0.32 [−0.48 to −0.15]) and taurine (−0.13 [−0.25 to −0.02]). The bile acids taurolithocholate (coefficient −0.05 [95% CI −0.07 to −0.02]) and taurodeoxycholate (−0.06 [−0.10 to −0.03]) were also present at higher levels in below average responders.

**Fig. 4:**
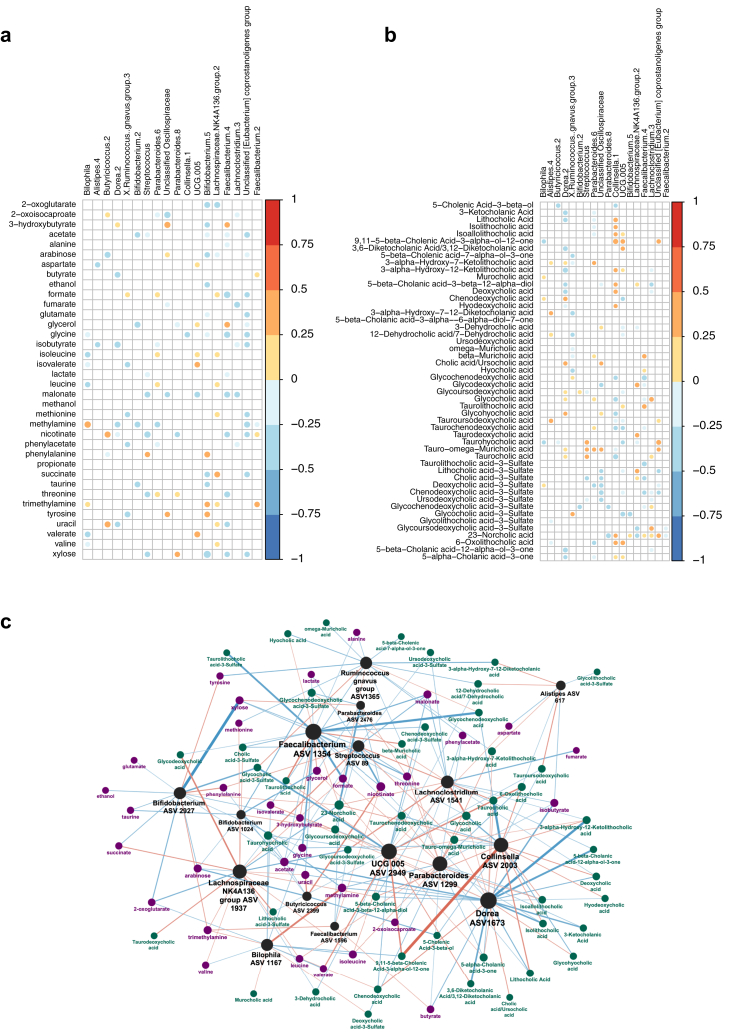
**Spearman correlation heatmaps and network analysis showing associations between gut microbiota and metabolites**. ASVs shown were identified as associated with above/below average response to vaccination and correlated with (a) metabolites identified from 1H NMR and (b) bile acids. Coloured circles signify correlations with q value <0.2. (c) Network analysis: ASVs are coloured black, metabolites identified in 1H NMR in purple, and bile acids in dark green. The size of the labels corresponds to the degree of the nodes, the colour and width of the edges corresponds to the correlation coefficient (the mapping function is “V” shaped: the stronger positive or stronger negative associations have a more intense colour/width, the weaker ones that are closer to zero are narrower/fainter).

### Integration of microbiome and metabolite data

Finally, correlation and network analyses were performed to determine metabolites that were associated with significant ASVs (Fig. 4). *Streptococcus* was positively correlated with the amino acid phenylalanine (r = 0.25); both were associated with below average response to vaccination in earlier independent analysis. *Streptococcus* was also correlated with taurocholic acid (r = 0.31), taurohyocholic acid (r = 0.32) and tauro-omega-muricholic acid (r = 0.26). *Bilophila* was positively associated with methylamine and trimethylamine (r = 0.37 & 0.23 respectively), and chenodeoxycholic acid (r = 0.22). The short chain fatty acid acetate was negatively correlated with *Streptococcus and Bifidobacterium* ASVs (r = −0.22 & −0.27 respectively), while valerate was negatively correlated with *Bilophila* (r = −0.30).

The network representation of the correlation matrices (Fig. 4c) highlighted Dorea ASV 1673, Faecalibacterium ASV 1354 and UCG 005 ASV 2949 as the highest degree ASVs, connecting to 30%, 28% and 27% of the significantly associated metabolites, respectively. Lachnospiraceae NK4A136 ASV 1937, UCG 005 ASV 2949 and Collinsella ASV 2003 drive 48% of the significant positive associations, with little overlap regarding the affected metabolites. The negative associations are primarily driven by Faecalibacterium ASV 1354 and Dorea ASV 1673, affecting 43% of the significant negative associations. Norcholic Acid, nicotinate and Glycocholic Acid are the highest degree metabolites, the three combined reaching over 73% of the ASVs in the network, with mixed association profiles. Acetate and trimethylamine are the most common positively and negatively associated metabolites, connecting to 30% and 26% of the ASVs, respectively.

## Discussion

To our knowledge, this study is the first to demonstrate a link between the metabolic function of the gut microbiota and immune responses to SARS-CoV-2 vaccination in a vulnerable group. These findings are particularly important because they pertain to an immunosuppressed cohort known to have diminished immunogenicity to vaccination, in whom rates of breakthrough SARS-CoV-2 infection are increased.

There is growing evidence from human^9^ and animal studies^10^^,^^11^ that the gut microbiota plays a role in modulating immune responses to vaccination, and the baseline composition of the gut microbiota has recently been shown to predict serological response to COVID-19 vaccines.^12^ There are a number of important differences between our study and that of Ng and colleagues, in which the majority of participants received CoronaVac, which is an inactivated virus vaccine, and a minority received BNT162b2 (mRNA vaccine). In our study the majority of participants received BNT162b2 and a minority received ChAdOx1 nCoV-19 (adenovirus vector vaccine). The two studies were carried out in different countries (Hong Kong and United Kingdom). Perhaps most importantly all participants in our study had IBD and were on infliximab, whereas the Ng study included predominantly healthy individuals, with fewer than 3% on any form of immunosuppressive therapy. Despite these differences, a common finding in both studies was that a *Parabacteroides* was associated with lower serological response to vaccination.

In our study *Bilophila* genus abundance was associated with higher serological response to SARS-CoV-2 vaccination. Elevated *Bilophila* abundance has been reported in treatment-naïve IBD patients^42^ and *Bilophila wadsworthensis* is associated with an enhanced pro-inflammatory T_H_1 response and development of colitis in *Il10*^*-/-*^ mice.^43^ Notably, vaccination with BNT162b2 leads to induction of virus-specific CD4+ T cell responses with a T_H_1 profile.^44^^,^^45^ It might be postulated that *Bilophila* is acting as a vaccine adjuvant in some IBD patients, engaging beneficial T-cell help to support the generation of antibodies against SARS-CoV-2.

Our data suggest that the gut microbiota derived metabolite trimethylamine (TMA) may be acting to ameliorate the attenuated vaccine-induced immune response seen in anti-TNF recipients. TMA is a breakdown product of gut microbial metabolism of the dietary constituents choline and carnitine.^46^ TMA is metabolised in the liver to trimethylamine N-oxide (TMAO), high plasma levels of which have been linked to the development of cardiovascular disease.^47^ Interestingly, the notion of TMAO as an immune sensitiser has recently been disclosed through its enhancement of the efficacy of cancer immunotherapy via promotion of CD8^+^ T cell-mediated immunity.^48^

We found several faecal metabolites, including phenylalanine, taurine and succinate, which are associated with reduced vaccine-induced serological responses. Faecal phenylalanine has been found at higher levels in IBD patients compared to healthy controls.^49^, ^50^, ^51^ Its metabolism has also been linked to the immune response to the bacillus Calmette-Guérin (BCG) vaccine.^52^ Succinate is found at higher levels in the gut of patients with ulcerative colitis relative to healthy controls.^53^^,^^54^ Succinate is also up-regulated in patients with fistulising Crohn's disease and is implicated in the epithelial to mesenchymal transition process.^55^ Paradoxically, succinate has also been shown to have anti-inflammatory activity via tuft cells in the small intestine.^56^ Another plausible mechanism linking gut microbiota function and immune response to vaccination is through the production of immune modulating SCFAs. A recent study has shown positive associations between SCFA levels after vaccination and higher serological response, although no differences in SCFAs were found at baseline.^13^ We found enrichment of the butyrate-producing Butyricicoccus ASV and the SCFA isobutyrate in patients with above average serological responses, although several ASVs with SCFA-producing potential were negatively associated with serological response.

We recognise several limitations in our study. Although our results are significant after correction for multiple testing, an *a priori* sample size calculation was not performed and the relatively small cohort and absence of external validation means that the findings would benefit from corroboration in larger populations. In our analysis we have accounted for confounding factors including age, gender, IBD subtype and IBD therapy, but we were not able to account for other factors known to influence the gut microbiome and metabolome such as diet. Microbiota analysis was performed by 16S rRNA amplicon sequencing rather than by shotgun metagenomics, meaning that we could explore differences in gut microbiome taxonomic profiles between high and low antibody responders down to ASV level, but not down to strain level. We have focussed our metabolomic analysis on faecal samples, and future studies analysing other biofluids including blood would be interesting. Furthermore, our immunological analysis is restricted to humoral immunity, and without T cell response data we are unable to draw conclusions on how the microbiota interact with vaccine-induced cell-mediated immunity. Finally, our study was observational and therefore we are not able to determine whether the identified microbiota or metabolomic associations are causative or mechanistically linked to vaccine response.

In conclusion, these results suggest a possible link between the composition and function of the gut microbiota and impaired serological response to SARS-CoV-2 vaccination in immunosuppressed individuals. These data imply that therapeutics targeted at modulating the microbiota, or even supplementation of beneficial metabolites, might be effective strategies to ameliorate diminished vaccine-induced immunogenicity in vulnerable groups.

## Contributors

JLA, BHM, TA and NP designed the study; JLA, AS, HP and MT collected the samples. JLA, NPD and JMB undertook microbiome analysis; JLA, ZL, BHM, VH and CS undertook metabolomic analysis; MLO and TK carried out the network analysis. JLA, BHM, NPD, ZL and NP wrote the first draft of the manuscript. All authors contributed to writing of and approved the final manuscript. JLA and NP have accessed and verified the data, and JLA and NP were responsible for the decision to submit the manuscript.

## Data sharing statement

Sequencing data from this study (in fastq-format) are publicly available for download at the European Nucleotide Archive (ENA) database using study accession number PRJEB52928 (http://www.ebi.ac.uk/ena/data/view/PRJEB52928). The datasets used and/or analysed during the current study are available from the corresponding author on reasonable request.

## Declaration of interests

Dr Saifuddin has received travel expense support from Janssen. Dr Lin reports non-financial support from Pfizer, non-financial support from Ferring, outside the submitted work. Dr. Kennedy reports grants from AbbVie, Biogen, Celgene, Celtrion, Galapagos, MSD, Napp, Pfizer, Pharmacosmos, Roche and Takeda, consulting fees from Amgen, Bristol-Myers Squibb, Falk, Janssen, Mylan, Pharmacosmos, Galapagos, Takeda and Tillotts, personal fees from Allergan, Celltrion, Falk, Ferring, Janssen, Pharmacosmos, Takeda, Tilllotts, Galapagos, and support for attending meetings from AbbVie, Falk and Janssen outside the submitted work. Prof. Sebastian reports grants from Takeda, Abbvie, Tillots Pharma, Janssen, Pfizer, Biogen and personal fees from Takeda, Abbvie, Janssen, Pharmacocosmos, Biogen, Pfizer, Tillots Pharma and Falk Pharma, outside the submitted work. Dr Hart reports payment or honoraria for lectures, presentations, speakers bureaus, manuscript writing or educational events from AbbVie, AZ, Atlantic, Bristol-Myers Squibb, Celltrion, Falk, Galapogos, Janssen, MSD, Napp Pharmaceuticals, Pfizer, Pharmacosmos, Shire and Takeda and Global Steering Committee for Genentech, support for attending meetings from Abbvie, Takeda and Janssen, and Participation on a Data Safety Monitoring Board or Advisory Board for AbbVie, AZ, Atlantic, Bristol-Myers Squibb, Galapogos, Janssen, Pfizer and Takeda. Prof. Lees reports a Future Leaders Fellow award from UKRI, personal consulting fees from Galapagos, Abbvie, Takeda, Pfizer, Janssen, Iterative Scopes and institutional consulting fees from Trellus Health, personal fees from Galapagos, Abbvie, Takeda, Pfizer, Janssen, 10.13039/100004330GSK, Gilead, Fresnius Kabi, Ferring and Dr Falk, and support for attending meetings from Galapagos, Abbvie, Takeda, Pfizer, Janssen, 10.13039/100004330GSK, Gilead, Fresnius Kabi, Ferring and Dr Falk. Dr. Goodhand reports grants from F. Hoffmann-La Roche AG, grants from Biogen Inc, grants from Celltrion Healthcare, grants from Galapagos NV, non-financial support from Immundiagnostik, during the conduct of the study. Prof. Ahmad reports grant funding from Pfizer to his institution to deliver this study, grants from Celltrion, Roche, Takeda, Biogen and Galapagos and honoraria for lectures from Takeda and Roche, outside the submitted work. Dr Powell has received research grant(s) from Bristol Myers Squibb outside the submitted work. Dr. Powell reports personal fees from Takeda, Janssen, Pfizer, Bristol-Myers Squibb, Abbvie, Roche, Lilly, Allergan, and Celgene, outside the submitted work; Dr. Powell has served as a speaker/advisory board member for Abbvie, Allergan, Bristol Myers Squibb, Celgene, Falk, Ferring, Janssen, Pfizer, Tillotts, Takeda and Vifor Pharma. The following authors have nothing to declare: Dr Alexander, Dr Ibraheim, Claire Bewshea, Rachel Nice, Dr Liu, Dr Mullish, Dr Danckert, Melissa Torkizadeh, Jesús Miguéns Blanco, Lauren A Roberts, Hemanth Prabhudev, Caroline Sands, Verena Horneffer-van der Sluis, Matthew Lewis, Professor Teare, Martin Olbei, Tamas Korcsmaros and Professor Marchesi.
