# Supplementary material for: The gut microbiota and metabolome are associated with diminished COVID-19 vaccine-induced antibody responses in immunosuppressed inflammatory bowel disease patients
Source: eBioMedicine. 2023 Jan 10;88:104430. doi: 10.1016/j.ebiom.2022.104430 (PMC9831064; doi:10.1016/j.ebiom.2022.104430)
Supplement: Supplementary material [file mmc1.docx]

**The gut microbiota and metabolome are associated with diminished COVID-19 vaccine-induced antibody responses in immunosuppressed inflammatory bowel disease patients**

Supplementary Material

Supplementary figure legend**:**

(a) Age distribution of participants with above (left) and below (right) average vaccine responses. The horizontal lines signify the median age in each group. (b) gender, (c) body mass index (BMI), (d) IBD subtype, (e) vaccine type, (f) use of immunomodulator (g) ethnicity, (h) comorbidity (presence of at least one of heart disease, diabetes mellitus, lung disease or kidney disease) distribution in above and below average vaccine responders. P values calculated using Mann-Witney test for continuous variables and Fisher’s exact test for categorical variables.
